# Multi-Fluorescence Real-Time PCR Assay for Detection of RIF and INH Resistance of *M. tuberculosis*

**DOI:** 10.3389/fmicb.2016.00618

**Published:** 2016-04-29

**Authors:** Jingfu Peng, Xiaoli Yu, Zhenling Cui, Wenfei Xue, Ziyi Luo, Zilu Wen, Minghua Liu, Danqing Jiang, Heping Zheng, Hai Wu, Shulin Zhang, Yao Li

**Affiliations:** ^1^State Key Laboratory of Genetic Engineering, Shanghai Engineering Research Center of Industrial Microorganisms, School of Life Science, Fudan UniversityShanghai, China; ^2^Department of Biotechnology, School of Biology and Pharmaceutical Engineering, Wuhan Polytechnic UniversityWuhan, China; ^3^Shanghai Key Laboratory of Tuberculosis, Medical School, Shanghai Pulmonary Hospital, Tongji UniversityShanghai, China; ^4^The Third People's Hospital of ShenzhenShenzhen, China; ^5^Haoding Technology Limited CompanyShenzhen, China; ^6^Department of Immunology and Medical Microbiology, School of Medicine, Shanghai Jiao Tong UniversityShanghai, China

**Keywords:** quantitative real-time PCR, *Mycobacterium tuberculosis*, drug resistance, rifampicin, isoniazid

## Abstract

**Background:** Failure to early detect multidrug-resistant tuberculosis (MDR-TB) results in treatment failure and poor clinical outcomes, and highlights the need to rapidly detect resistance to rifampicin (RIF) and isoniazid (INH).

**Methods:** In Multi-Fluorescence quantitative Real-Time PCR (MF-qRT-PCR) assay, 10 probes labeled with four kinds of fluorophores were designed to detect the mutations in regions of *rpoB, katG, mabA*-*inhA, oxyR*-*ahpC*, and *rrs*.

The efficiency of MF-qRT-PCR assay was tested using 261 bacterial isolates and 33 clinical sputum specimens. Among these samples, 227 *Mycobacterium tuberculosis* isolates were analyzed using drug susceptibility testing (DST), DNA sequencing and MF-qRT-PCR assay.

**Results:** Compared with DST, MF-qRT-PCR sensitivity and specificity for RIF-resistance were 94.6 and 100%, respectively. And the detection sensitivity and specificity for INH-resistance were 85.9 and 95.3%, respectively. Compared with DNA sequencing, the sensitivity and specificity of our assay were 97.2 and 100% for RIF-resistance and 97.9 and 96.4% for INH-resistance. Compared with Phenotypic strain identification, MF-qRT-PCR can distinguish 227 *M. tuberculosis* complexes (MTC) from 34 Non-tuberculous mycobacteria (NTM) isolates with 100% accuracy rate.

**Conclusions:** MF-qRT-PCR assay was an efficient, accurate, reliable, and easy-operated method for detection of RIF and INH-resistance, and distinction of MTC and NTM of clinical isolates.

## Introduction

Worldwide prevalence, high misdiagnosis rate, high treatment cost, and serious side-effects of drug made MDR-TB a critical problem for global public health (WHO, 2012). Therefore, rapid and accurate assays for resistance to first line anti-TB drugs (RIF and INH being the most significant) are decisive in choosing drugs when starting therapy.

Conventional drug susceptibility testing (DST) is the current “gold standard” for assessment of TB Drug Resistant. But failure of rapid diagnosis of DST leads to rising mortality, secondary resistance, and transmission of drug resistant-TB cases.

Alternatively, molecular assays provide faster turnover time while maintaining high sensitivity and specificity. Quantitative real-time polymerase chain reaction (qRT-PCR) is considered as one of the most widely applied molecular assays for diagnosis of drug resistant tuberculosis (Cuevas-Córdoba and Zenteno-Cuevas, 2010). It can accurately discriminate single nucleotide polymorphism (SNP) with low risk of contamination and had the shortest turn-around time (TAT) so far. qRT-PCR assays have been applied to the detection of RIF and INH resistances (García de Viedma et al., 2002; van Doorn et al., 2003; Marín et al., 2004; Helb et al., 2010) using at most two color probes so far. Most of these assays only detected mutations in segments of *rpoB* related to RIF and/or in segments of *katG and inhA* related to INH resistances. And some similar assays distinguished *Mycobacterium tuberculosis* complex (MTC) from Non-tuberculous mycobacteria (NTM) at the same time (Wada et al., 2004; Ramirez et al., 2010). Several commercial MTC/DST diagnostic kits based on qRT-PCR assay have appeared and obtained regulatory approval (WHO, 2012; Food and Drug Administration, 2015) including Xpert MTB/RIF (Cepheid) (Boehme et al., 2010), Fluorotype MDRTB (Hain Lifescience) (Pierce et al., 2003; Rice et al., 2012), RealTi*m*e MDR TB (Abbott Molecular) (Chen et al., 2015), BDProbeTec (Becton Dickinson) (UNITAID, 2015) and MeltPro Drug-Resistant TB RIF, and Drug-Resistant INH kits(Xiamen Zeesan Biotech) (Hu et al., 2014). While most of these kits can be automated and high-throughput detection of mutations, they still have shortcomings such as high cost, limited detection sites.

In the present study, we designed an improved MF-qRT-PCR assay that uses 10 probes for the detection of drug-resistant mutations in the 81-bp hot-spot region of *rpoB*, codon 315 of *katG*, −15 position in the promoter of *mabA*-*inhA*, −6 ~ −47 positions in the intergenic region of *oxyR*-*ahpC*, and 451 ~ 471 positions in *rrs* to distinguish MTC from NTM simultaneously. We evaluated this assay using 261 bacterial isolates and 33 sputum specimens from three cities (Shenzhen, Wuhan, and Shanghai) in China, compared to DNA sequencing and phenotypic DST results.

And we compared the advantages and disadvantages of MF-qRT-PCR assay with other diagnosis methods in Discussion.

## Materials and methods

### Bacterial isolates and clinical specimens

Two hundreds and sixty-one bacterial isolates of TB patients were used in this study. Among these, 150 isolates (collected at the Wuhan Medical Treatment Center from August 2009 to March 2010) were routinely cultured with egg-based Löwenstein-Jansen medium at 37°C, 91 isolates (collected at the Shenzhen Third People's Hospital from January 2011 to November 2011) and 20 isolates (collected at Shanghai Pulmonary Hospital) were selected from the cultures grown in Bactec MGIT 960. Thirty-three clinical sputum specimens of TB suspects were selected randomly from Shenzhen Third People's Hospital. H37Rv strain was a gift from State Key Laboratory of Genetic Engineering, Institute of Genetics, School of Life Science, Fudan University and was served as the wild-type *Mycobacterium tuberculosis* Complex (MTC) control.

DST, sequencing, and MF-qRT-PCR assay were performed on all bacterial isolates. Clinical sputum specimens were divided into two portions. One portion of each sputum sample was cultured using Bactec MGIT 960. The other portion was treated to perform MF-qRT-PCR, and was analyzed by TB quantitative PCR kit (Qiagen, Germen).

### MF-qRT-PCR

#### Sample treatment and DNA extraction

DNAs of culture isolates were re-suspended into 100 μl lysis buffer which composed of 50 mmol/L Tris-HCl(Ph8.0), 2 mM EDTA(Ph8.0),100 mmol/L NaCl, heated at 100°C for 15 min, then centrifuge at 5000 × g for 1 min, and discard the sediment. DNA was re-suspended in miliQ H_2_O as PCR template or stored at −20°C until it was used.

In order to extract DNAs of clinical sputum specimens, 2–3 volumes of 4% NaOH were added to the sputum specimens. The mixture was agitated at 140 rpm for 30 min at 37°C. Then 1 ml completely liquefied mixture were added to a new centrifuge tube, centrifuged at 13,000 rpm for 10 min, and discarded supernatant. Added 1 ml normal saline into the sediment, centrifuged at 13,000 rpm for 10 min, discarded supernatant. Then followed treatments of culture isolate specimens mentioned above.

#### Real-time PCR

##### Probes design

Ten dually labeled probes and five pairs of primer were designed to detect key mutations at 40 positions in four gene regions associated with RIF and INH resistance and conserved region distinguished MTC from NTM (Table 1).

Table 1**Primers and Probes designed and used in this study**.

**Table:** 

**Primer**	**Target**	**Reation^a^**	**Conc (μM)**	**Sequence(5′-3′)**	**Product size (bp)**
**REAL-TIME PCR PRIMERS**					
rpoB-FP	*rpoB*	A,B	0.6	GGTCGCCGCGATCAAGGA	130
rpoB-RP		A,B	0.6	CTCACGTGACAGACCGCCG	
katG-FP	*katG*	B	0.3	GATGGGCTTGGGCTGGAA	131
katG-RP		B	0.3	AGCCGTACAGGATCTCGAGGAA	
inhA-FP	promoter of *mabA-inhA*	B	0.3	GGAAATCGCAGCCACGTTAC	96
inhA-RP		B	0.3	TTCAGTGGCTGTGGCAGTCA	
rrs-FP	16S rRNA	B	0.3	CCTTCGGGTTGTAAACCTCTTTC	132
rrs-RP		B	0.3	GGACAACGCTCGCACCC	
ahpC-FP	intergenic region of *oxyR-ahpC*	C	0.3	CGGCGATGCCGATAAATATG	101
ahpC-RP		C	0.3	TCATCAAAGCGGACAATGCA	
**SEQUENCING PRIMERS**					
rpoB-CFP	*rpoB*		0.5	CGACGACATCGACCACTTC	501
rpoB-CRP			0.5	GGCGGTCAGGTACACGAT	
katG-CFP	*katG*		0.5	GGCGATGAGCGTTACAGC	670
katG-CRP			0.5	CCAAGGTATCTCGCAACGG	
inhA-CFP	promoter of *mabA-inhA*		0.5	CCTCGCTGCCCAGAAAGGGA	248
inhA-CRP			0.5	ATCCCCCGGTTTCCTCCGGT	
ahpC-CFP	intergenic region of *oxyR-ahpC*		0.5	GACCGGCTTCCGACCA	472
ahpC-CRP	intergenic region of *oxyR-ahpC*		0.5	AACTCGTCATTGAGCTTGCTG

**Table:** 

**Probe**	**Target^a^**	**Reation^b^**	**Conc(μM)**	**Sequence(5′–3′)**	**5′Fluorescence dye**	**Detective wavelength (nm)**	**3′Quenching dye**
rpoB-TW-A	*rpoB* 509–514	A	0.2	GAATTGGCTCAGCTGGCTG	CY5	667	BHQ3
rpoB-TW-B	*rpoB* 515–520	A	0.1	ATGGACCAGAACAACCCG	ROX	602	BHQ2
rpoB-TW-C	*rpoB* 520–524	B	0.3	GTCAACCCCGACAGCGG	CY5	667	BHQ3
rpoB-TW-D	*rpoB* 524–529	A	0.6	GTTGACCCACAAGCGCCG	HEX	556	eclipse
rpoB-TW-E	*rpoB* 529–533	A	0.6	CAGCGCCGACAGTCG	FAM	520	eclipse
katG-TW	*katG* 313–318	B	0.1	ATCACCAGCGGCATCG	FAM	520	eclipse
inhA-TW	*mabA-inhA* −8~–24	B	0.1	GCGGCGAGACGATAGGT	ROX	602	BHQ2
ahpC-TW-A	*oxyR-ahpC* −2~–20	C	0.3	CTTCACGGCACGATGGAAT	CY5	667	BHQ3
ahpC-TW-B	*oxyR-ahpC* −28~–49	C	0.3	TGTGATATATCACCTTTGCCTG	HEX	556	eclipse
rrs-TW	rrs(16S rRNA)	B	0.6	AGGTCCGGGTTCTCTCGGATT	HEX	556	eclipse

a Negative numbers in designations indicate positions of mutations located before the start codon of the gene.

b *A, B, and C, the three multi-fluorescence real-time PCR reactions*.

**Figure 1:**
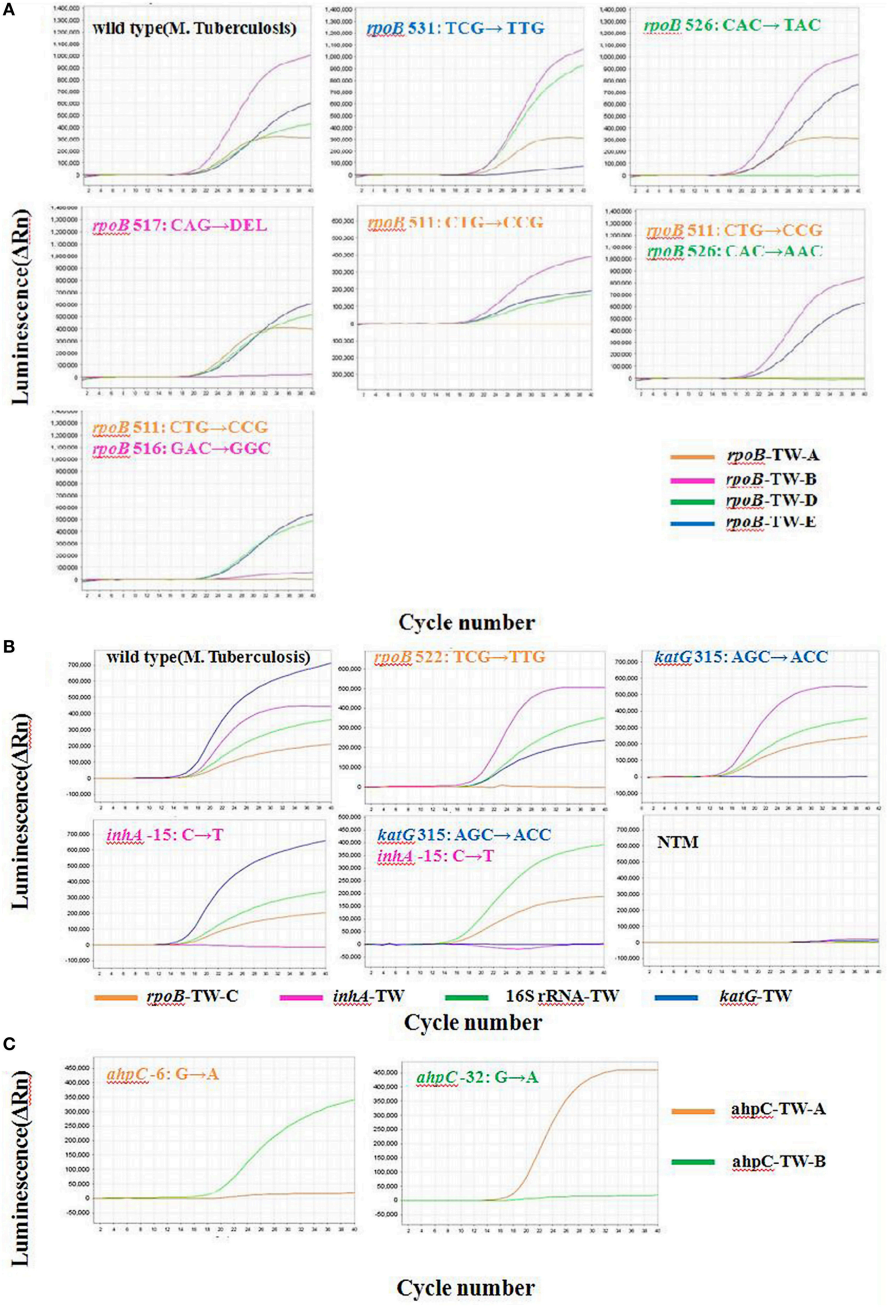
**Analysis of DNAs from Mycobacteria with 10 TaqMan probes by multi-fluorescence real-time PCR**. **(A)** Results of reation I **(B)** Results of reation II **(C)** Results of reation III.

Four fluorescence (FAM, HEX, ROX, CY5/TAMRA) were labeled at the 5′end of probes to simultaneously detected in a single PCR reaction (Figure 1).

We determined the probe coverage according to the position and frequency of the key drug resistant mutations described in TB Drug Resistant Mutation Database (https://tbdreamdb.ki.se/Info/) and our own sequencing results. Specifically, probe rpoB-TW-A covers mutations in *rpoB* codon 509–514, probe rpoB-TW-B in condon 515–520; probe rpoB-TW-C in condon520–524; probe rpoB-TW-D in condon 524–529; probe rpoB-TW-E in condon 529–533. And probe katG-TW covers mutations at *katG* condon315; probe inhA –TW for mutation at −15 position in *mabA*-*inhA* promoter region; probe ahpC-TW-A for mutations from −2 to −20 positions in intergenic region of *oxyR*-*ahpC*, probe ahpC-TW-B for mutations from −28 to −47 positions in intergenic region of *oxyR*-*ahpC.* In addition, probe rrs -TW was designed to distinguish MTC from NTM, according to *rrs* sequence of Mycobacterium strains in the NCBI database (Table 1, Figure S1).

Three PCR reactions were required to diagnose samples' RIF and INH resistance. PCR mixture contained: 10 × PCR buffer 2 μl, dNTP (each 2.5 μM) 1.6 μl, MgCl_2_ (25mM) 0.8 μl, Taq(5U/ul) 0.2 μl, primers, and probes (Optimal concentrations of primers and probes for each PCR reaction are shown in Table 1), extracted DNA template 1 μl, miliQ H_2_O was added to make up the whole PCR mixture volume to 20 μl. Final concentrations of probes were different, in order to get the equivalent fluorescence signal level. Cycling parameters were denatured at 94°C for 2 min, followed by 40 cycles of amplification at 95°C for 15 s, 62°C for 1 min. A negative control which ddH_2_O replace the DNA sample and a positive control (DNA extraction of H37Rv) were included for each real-time PCR assay.

#### Results analysis

Firstly, when rrs probe signal was negative, template is determined as NTM, positive signal means MTC. Secondly, signals of the other nine drug resistant probes were investigated. Positive signals of all the five RIF probes (rpoB-TW-A ~rpoB-TW-E) represented no mutation in the 81 bp core region of *rpoB*, meaning that this sample was RIF-sensitive, otherwise means the sample is RIF-resistant. Similarly, the sample was INH-sensitive when probes katG-TW, inhA-TW, ahpC-TW-A, and ahpC-TW-B were all positive. Otherwise means INH-resistant (Table S1).

#### Analytical sensitivity and specificity

Reactions were performed with serially diluted genomic DNAs (gDNA; range, 5.0 × 10^5^–5.0 × 10^0^ copies per reaction mixture) of H37Rv. Concentration of each gDNA was determined by an SMA1000 UV spectrophotometer (Merintion, CN).

In order to investigate the specificity of MF-qRT-PCR, DNAs extract from H37Rv,BCG,E.coli, clinical strains of *M. tuberculosis, M. bovis*, and NTM were used as real time - PCR template.

#### Statistical analysis

Data was categorized using Microsoft EXCEL 2010 and processed using the SPSS software package, version 19.0. Specificity, sensitivity, positive and negative predictive values and 95% confidential interval (CI) of MF-qRT-PCR assay were calculated with Graph Pad Prism 6.

### Sequencing

Following fragments were amplified and sequenced: 501 bp segment of *rpoB*, 670 bp of *katG*, 248 bp of *inhA* promoter region, 472 of intergenic region of *oxyR-ahpC*. Sequencing primer sequences were presented in Table 1. PCR conditions were denaturation at 94°C for 10 min, followed by 35 cycles of amplification at 94°C for 30 s, 60°C for 30 s, 72°C for 30 s. Primers were synthesized by JieLi Bio Co. (China). PCR mixtures were prepared using 2 × GoldStar Best MasterMix (CWBio Co., China). Sequence data were assembled and analyzed by BioEdit software.

### Drug susceptibility testing (DST)

Proportion method was performed on DSTs use the following drug concentrations in the modified Löwenstein-Jensen medium: RIF, 40.0 μg/ml; INH, 0.2 μg/ml (Zhang et al., 2007).

### Identification of isolates

261 MTC and NTM discrimination Culture isolates from Wuhan previously identified by conventional biochemical methods were confirmed by 16S rRNA gene sequencing. Strains isolates from Shenzhen and Shanghai were discriminate as MTC or NTM by Bactec MGIT 960.

## Results

### Characterization of clinical isolates (strain identification and conventional DST results)

A total of 261 isolates were collected (Table 2). Among these, 227 isolates (87.0%) were MTC, 34 (13.0%) isolates were NTM. Among MTC, Phenotypic DST showed that 50 (22.0%) were pan-susceptible and177 (78.0%) resistant to at least one drug. Specifically, 14 isolates (6.17%) were only resistant to rifampin and 29 isolates (12.8%) merely to isoniazid, and combined resistance to both drugs (MDR-TB) was recorded in 134 strains (59.0%).

**Table 2:** **Locality, species, and drug resistance (detected by three methods) of samples in this study**.

**A**					**B**			
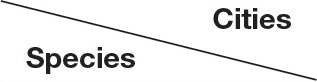	**SZ^a^**	**WH^b^**	**SH^c^**	**SUM**	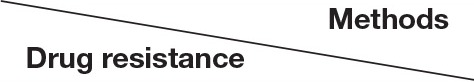	**DST^f^**	**Sequencing**	**Multi-fluorescence real-time PCR**
MTC^d^	76	131	20	227	pan-sensitive	50	65	65
NTM^e^	15	19	0	34	SDR^g^(RIF-resistant)	14	19	19
SUM	91	150	20	261	SDR (INH-resistant)	29	18	22
					MDR^h^	134	125	121
					SUM	227	227	227

a SZ, Shenzhen;

b WH, Wuhan;

c SH, Shanghai;

d MTC, Mycobacterium tuberculosis Complex;

e NTM, Non-tuberculous Mycobacteria;

f DST, drug susceptibility testing;

g SDR, Single-drug Resistant;

h MDR, Multi-drug Resistant.

### Identification of mutations in clinical isolates (sequencing results)

Table S2 shows detailed information about the mutations that detected in *rpoB* 81-bp core region. Altogether, 63.4% (144/227 isolates) of the MTC isolates, or 97.3% (144/148 isolates) of the RIF-resistant isolates, harbored mutations in 14 codons of the 81-bp core region of *rpoB*. The most frequently mutated *rpoB* codons were codons 531, 526, 516, 511, and 533 with mutation frequencies of 53.5, 20.8, 13.2, 3.47, and 3.47%, respectively. These five mutations can emerge independently. No mutations in *rpoB* were detected in the 79 RIF-sensitive isolates.

A total of 63.0% (143/227) of MTC, or 87.7% (143/163) of INH-resistant strains had mutations in one or more of three regions (Table S3). The most frequent mutation site was *katG315*; 90.0% (99/110). 15.1% (25/166) of the INH-resistant strains harbored mutations in the promoter region of *mabA-inhA*. Most of the mutations appear at −15 positon with a ratio of 84% (21/25). 16.3% (27/166) of the INH-resistant strains had mutations in intergenic region of *oxyR-ahpC*. Most mutations in these region scattered at *ahpC*-6, *ahpC*-9, *ahpC*-10,*ahpC*-12,*ahpC*-17,*ahpC*-32, and *ahpC*-39 with similar ratio.

Taken together, mutations in the 81-bp core region of *rpoB* and mutation sites in three isoniazid resistance-conferring regions identified 97.3% of the RIF-resistant isolates and 87.7% of the INH-resistant isolates.

### Assess sensitivity and specificity of MF-qRT-PCR

Serial dilutions of *M. tuberculosis* H37Rv DNA were detected employing MF-qRT-PCR. Results revealed the sensitivity of this assay. For rrs-TW probe, 5.0 × 10^0^ copies/reaction of H37Rv DNA could be identified effectively (Figure S2).

Signals of 16S rRNA probes were negative, when H_2_O, DNAs extracted from clinical strains of NTM as well as *E. coli* were used as real time—PCR template. On the other hand, we got positive results of 16S rRNA signals when detecting H37Rv, clinical strains of *M. tuberculosis, M. bovis*, and BCG. Therefore, specificity of this method for the *M. tuberculosis* complex was quite prominent.

### qRT-PCR evaluation of MF-qRT-PCR assay

Two hundred and twenty seven DNAs extractions of MTC were analyzed using DST, DNA sequencing, and MF-qRT-PCR. Compared to Phenotypic DST results, for RIF-resistance, the sensitivity of MF-qRT-PCR was 94.6% (91.0–98.2%), specificity was 100.0%, positive predictive value was 100.0%, negative predictive value was 90.8%(84.7–96.9%). For INH-resistance, the sensitivity was 85.9% (80.6–91.2%), specificity was 95.3% (90.1–100.0%), positive predictive value was 97.9% (95.6–100.0%), negative predictive value was 72.6% (63.1–82.2%) (Table 3). Compared to DNA sequencing results, for RIF-resistance, the sensitivity was 97.2% (94.5–99.9%), specificity was 100.0%, positive predictive value was 100.0%, negative predictive value was 95.4%(91.0–99.8%). For INH-resistance, the sensitivity was 97.9% (95.6–100.0%), specificity was 96.4% (92.5–100.0%), positive predictive value was 97.9% (95.6–100.0%), negative predictive value was 96.4% (92.5–100.0%) (Table 3).It suggested that the results of MF-qRT-PCR were quite consistent with the other two “gold standard” methods, especially for DNA sequencing.

**Table 3:** **Sensitivities and specificities of multi-fluorescence real-time PCR compared to DST and DNA-sequencing results**.

**Group**	**DST results**		**Sensitivity (%)**	**Specificity (%)**	**PPV (%)**	**NPV (%)**
	**Resistant**	**Susceptible**				
**RIF**						
Mutant	140	0	94.6	100.0	100.0	90.8
Wild type	8	79				
**INH**						
Mutant	140	3	85.9	95.3	97.9	72.6
Wild type	23	61				
**Group**	**DNA-sequencing results**		**Sensitivity (%)**	**Specificity (%)**	**PPV (%)**	**NPV (%)**
	**Resistant**	**Susceptible**				
**RIF**						
Mutant	140	0	97.2	100.0	100.0	95.4
Wild type	4	83				
**INH**						
Mutant	140	3	97.9	96.4	97.9	96.4
Wild type	3	81				

MF-qRT-PCR results of several typical mutants were shown in Figure 1. Figure 1 showed when the probes of MF-qRT-PCR assay detected their target DNA segments (wild type, with no typical mutants), apparent fluorescence signals were released, and “S” shape amplification curves of PCR appeared. When the probes detected target DNA segments (with at less one mismatch base), no significant signal would be released, and amplification curves would be flat. It means that the probes of MF-qRT-PCR assay can distinguish typical mutants in regions of *rpoB, katG, mabA*-*inhA, oxyR*-*ahpC*, and *rrs* from wild types very easily and clearly.

Additional 34 NTM isolates were analyzed along with the 227 MTC isolates. MF-qRT-PCR can distinguish the NTM isolates from MTC isolates with 100% accuracy rate.

Clinical sputum sample cultured, quantitative PCR and MF-qRT-PCR assay results all showed that 16 sputum specimens were TB positive and 17 were TB negative. All 16 TB positive sputum specimens were RIF and INH sensitive using MF-qRT-PCR assay and sequencing.

## Discussion

### Drug-resistant mutation

Molecular mechanism of resistance to anti-TB drugs is becoming clearer. Several studies have demonstrated that 90–95% of isolates with rifampin resistance have mutations within the 81-bp core region (codons 508–533) of *rpoB*, (Alcaide and Telenti, 1997), with emphasis on the codons 516, 526, and 531(Escalante et al., 1998),or codons 531, 526, 516, 533, 513 were the most emerged mutations(Luo et al., 2010). Mutations at codon 315 of *katG*, −15 position of the promoter of *mabA*-*inhA* and −6 ~ −47 positions of intergenic region of *oxyR*-*ahpC* are considered to be responsible for 80–90% of strains resistant to INH (Cockerill et al., 1995; Slayden and Barry, 2000). Presence of these mutations indicates RIF and INH resistance. Our sequencing result approved these conclusions. Mutations in *rpoB* 3 isoniazid resistance-conferring regions identified 97.3% of the RIF-resistant isolates and 87.7% of the INH-resistant isolates.

### Molecular DST methods of TB

Various genotypic molecular methods, especially nucleic acid–amplification technologies, arose in recent years(García de Viedma, 2003; Cuevas-Córdoba and Zenteno-Cuevas, 2010; Abebe et al., 2011), which included conventional sequencing (Martin et al., 2006), pyrosequencing (Jureen et al., 2006), high-resolution thermal melt analysis (Hoek et al., 2008; Ramirez et al., 2010), denaturing gradient gel electrophoresis (DGGE) (McCammon et al., 2005), solid-phase hybridization-line probe assays, INNO-LiPA Rif (Quezada et al., 2007). TB assay (Innogenetics, Belgium), GenoType MTBDR Plus assay (HainLifescience, Nehren, Germany) (Brossier et al., 2009), molecular beacon-based automated platform Xpert MTB/RIF (Boehme et al., 2010), and digital PCR (Pholwat et al., 2013). Two main classes of real-time PCR methods had been applied to detect mutations related to drug-resistance. In the first class, mutation is detected by melting curve analysis. Three major assays, fluorescence resonance energy transfer (FRET) probe melting curve analysis (Saribas et al., 2005), high-resolution melting curve (HRM) analysis (McCammon et al., 2005; Pietzka et al., 2009; Ramirez et al., 2010), and probe-based melting curve analysis technologies(Luo et al., 2011) (including unlabeled probes, dually labeled probes, and sloppy molecular beacons), have been applied to detect drug-resistant mutations in *M. tuberculosis.* Some commercial diagnostic kits are based on melting curve analysis including Fluorotype MDRTB(Hain Lifescience) and Drug-Resistant TB RIF and Drug-Resistant INH kits(Xiamen Zeesan Biotech). In the second class, fluorescent signals are generated by hybridization of probes to the target sequences at the end of each PCR cycle. The fluorogenic 5′exonuclease probe-(TaqMan) (Hillemann et al., 2006), minor groove binder probe- (MGB) (Wada et al., 2004) and molecular beacon-based mutation distinguish assays (Banerjee et al., 2010) belong to this class. Commercial diagnostic kits Xpert MTB/RIF (Cepheid), RealTi*m*e MDR TB (Abbott Molecular), and BDProbeTec™ (Becton Dickinson) were based on this class method. Among these methods, the TaqMan probe coupled with the real-time PCR technology is one of the most accurate, reliable, and cost-effective nucleic acid–amplification methods.

In this study, we have developed MF-qRT-PCR assay which used probes with four fluorescent labels for analysis of RIF and INH resistance, by detecting drug-resistant mutations in the 81-bp hot-spot region of *rpoB*, codon 315 of *katG*, −15 position in promoter of *mabA*-*inhA*, −6 ~ −47 positions in intergenic region of *oxyR*-*ahpC*. And we can also distinguish MTC from NTM at the same time by detecting mutations between 451~471 positions in gene rrs which transcript 16S rRNA.

### The advantage of MF-qRT-PCR

Comparing with the probe based (TaqMan, MGB, molecular beacon) qRT-PCR method reported before, MF-qRT-PCR had several advantages.

Firstly, detection ranges of MF-qRT-PCR were wider than other probes based qRT-PCR before(Torres et al., 2003; Lin et al., 2004; Yesilkaya et al., 2006; Zenteno-Cuevas et al., 2012). For RIF-resistance, MF-qRT-PCR detected whole 81 bp core region in rpoB gene, instead of some mutation in this region. For INH-resistance, MF-qRT-PCR not only detected mutations in katG and promoter of *mabA*-*inhA*, but also in intergenic region of *oxyR*-*ahpC*. These improvements increased sensitivity of drug resistance assay, especially for INH-resistance.

Secondly, comparing with (Gomez et al., 2010), data analysis and result determination of MF-qRT-PCR were easier and clearer. No need to calculate and compare the CT values between positive control and negative control, just directly distinguish positive and negative result by threshold line.

Thirdly, more fluorescence dyes were employed [four-color instead of single or two-color(Torres et al., 2003; Lin et al., 2004; Yesilkaya et al., 2006; Gomez et al., 2010; Zenteno-Cuevas et al., 2012)] in our study. So, MF-qRT-PCR analysis can reduce the amount of the PCR reactions, decrease the differences between PCR tubes, and increase the convenience of operation.

Fourthly, the sensitivity and specificity of MF-qRT-PCR method were equivalent to the 3′-minor groove binder(MGB) probes-based method(van Doorn et al., 2003; Wada et al., 2004; Madania et al., 2012) and the cost is about a half of the latter.

When compared to Xpert MTB/RIF(Boehme et al., 2010), MF-qRT-PCR method not only identified *rpoB* 81 bp core region of mutation (RIF-resistant) but also detected *katG* and *inhA* (INH- resistance). Instead of the flanking sequence of *rpoB* core region that XpertMTB/RIF adapt to, we designed MTC identification probe targeting the special region within the 16S rRNA- coding gene (*rrs*). In this region, the MTC sequences are highly dissimilar to the sequences of NTM. Therefore, we can gain drug susceptibility (RIF and INH resistance) results and distinguish MTC from NTM in a single test. As the genome of MTC has more copies of 16S rDNA than *rpoB*, rrs(16S-rDNA) probe will have higher assay sensitivity than probes targeting *rpoB*.

Comparing with melt-curve method (Pietzka et al., 2009; Ramirez et al., 2010; Luo et al., 2011), MF-qRT-PCR assay had advantages too.

Firstly, as outlined above, MF-qRT-PCR assay can detect more genetic loci (such as the intergenic region of *oxyR*-*ahpC*) than previous studies. So the sensitivity of INH-resistance detection was increased.

Secondly, MF-qRT-PCR assay had easier and clearer result criteria, because in every mutant position, operator can easily distinguish “S” shape amplification curves (means wild type, no mismatch base existed) from flat line (means mutant, at less one mismatch base existed), as Figure 1 showed, explained in Results part). In addition, melt-curve method (Pietzka et al., 2009) had to compare the melting peak temperature between test specimens and standard wild-type specimens. In another study (Ramirez et al., 2010), researcher couldn't distinglish A->T transversion SNPs from wild type in HRM graph displayed in normalized mode, so additional amplification curve assay had to be used to detect Asp516Val in *rpoB.* That increases the complexity of analysis.

It was more subjective than the former and requires more experimental experience to operate, especially when multiple mutations emerge simultaneously in one reaction or fluorescent signal is weak.

Finally, the whole testing progress of MT-qRT-PCR can be finished in 2.5 h. It is faster than conventional DST and most of the molecular drug-resistance assays.

## Limitations and improvement

Compare to sequencing result, MF-qRT-PCR got four false negative cases in detecting RIF resistance (Table 3). We believed that these four were mixed infected samples (contained both wild type and drug-resistant mutant strains). Therefore, wild type probes used in MF-qRT-PCR can match the wild type templates in mix type samples, generate significant signals of amplification. Mutant templates in mix type samples were overshadowed. These lead to wild type results (false negative cases). At the same time, three false negative and three false positive cases were found in detecting INH resistance (Table 3). Cause of three false negative cases was the sequences of probe inhA-TW which targeted to *mabA*-*inhA* −8 ~−24 positions (Table 1). The three false negative cases all have mutant at *mabA*-*inhA* −8 position which was at the 3′end of probe inhA-TW. It means *mabA*-*inhA* −8 were not in the best position of probe to distinguish SNP (Figure S1). The reason of these three false positive should be the low emission efficiency of CY5 labeled probe (ahpC-TW-A) which gave out weak amplified signal and misjudge wild type *ahpC* loci to mutant.

Compared with DST results, MF-qRT-PCR got increased false negative case (eight in detecting RIF, 23 in detecting INH resistance) (Table 3). These were because of the existence of unknown drug resistant loci and mechanisms.

MF-qRT-PCR needs real-time PCR instrument with multi-waved channel (no less than four channel). Because of the rapid spreading of advanced real-time PCR instrument in China resent years, instrument limitations to MF-qRT-PCR were greatly relieved.

Our method had difficulty in detecting mixed strains (both wild type and drug-resistant mutant) directly, such as Xpert MTB/RIF. All probes we used are wild-type probes, so if wild type templates and mutant templates were both in the specimens, test result would be positive, which covered the presence of the mutant template. However, if we could control the concentration of the initial template before PCR reaction, the CT value of the pure wild-type template will be significantly smaller than the mix infected template. So if we calculated the ΔCt between pure wild-type and test sample, and set an appropriate CUTOFF value, we could semi-quantitatively distinguish wild-type infection from mixed infection.

Low number of positive and drug-resistance clinical specimens and the lack of susceptibility results of these samples prevented us from drawing conclusions about performance of MF-qRT-PCR according to clinical specimens. Further studies are needed to validate MF-qRT-PCR in these specimens.

Sequence of probe inhA-TW need to be optimized to target to mabA-inhA −4 ~−20, so that mutant at mabA-inhA −8, −15, −17 positions can be detected at the same time.

It's easy to increase a probe that specifically recognizes most species of Mycobacterium using this approach, so that it can cooperate with rrs (16S rRNA) probe to distinguish MTC, NTM, and other bacteria succinctly and accurately. Likewise, probes targeting other genes or regions associated with other anti-TB drugs could be added to this assay without technical limitations.

## Conclusions

In summary, we have developed a widely applicable MF-qRT-PCR assay to detect RIF and INH-resistant mutations in *M. tuberculosis* and distinguish MTC from NTM in 2.5 h. This improved approach has been proven to be efficient, accurate, reliable, and easy-operated. Further studies are required to optimize this method and evaluate its performance on clinical sputum and other types of specimens.

## Author contributions

SZ, XY, and YL designed experiments; JP, XY, ZC, WX, ZL, ZW, ML, HZ, DJ, and HW carried out experiments; SZ, XY, YL, and JP analyzed experimental results; WX, ZL, ZW, and HZ analyzed sequencing data and developed analysis tools; JP, XY, SZ, and YL wrote the manuscript.

### Conflict of interest statement

The authors declare that the research was conducted in the absence of any commercial or financial relationships that could be construed as a potential conflict of interest.
