# Supplementary material for: Multi-Fluorescence Real-Time PCR Assay for Detection of RIF and INH Resistance of M. tuberculosis
Source: Front Microbiol. 2016 Apr 29;7:618. doi: 10.3389/fmicb.2016.00618 (PMC4850356; doi:10.3389/fmicb.2016.00618)
Supplement: Supplementary file 1 [file DataSheet1.DOCX]

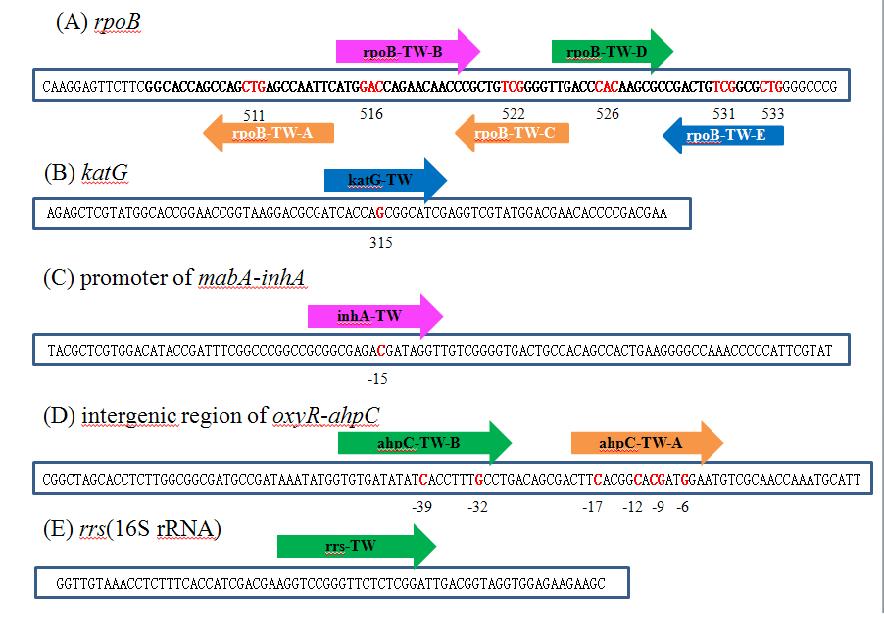


Figure S1. (A) Schematic representation of the *rpoB* 81bp core region and 5 labeled probes.(B) Schematic representation of the *katG* and 1 labeled probes. (C) Schematic representation of the promoter of *mabA-inhA* and 1 labeled probes. (D) Schematic representation of the intergenic region of *oxyR-ahpC* and 2 labeled probes. (E) Schematic representation of *rrs*(16S rRNA) and 1 labeled probes.


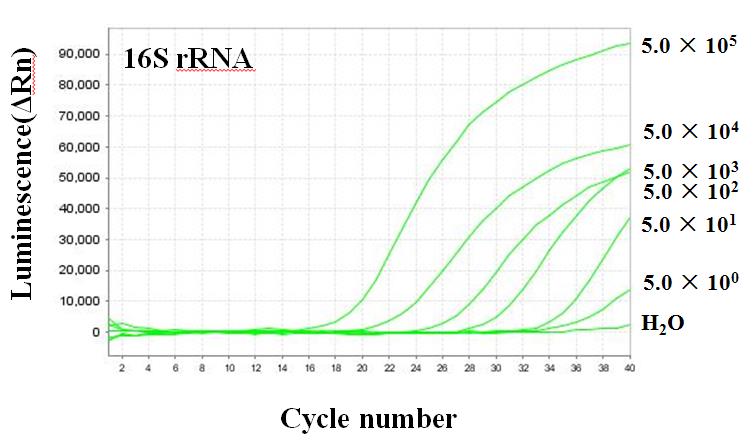


Figure S2. Amplification curves of probe *rrs*-TW with *M.tuberculosis* H37Rv DNA from 5.0×10^5^ to 5.0×10^0^ copies per reaction mixture.

| Table S1. Algorithm for detection of mono resistance and MDR TB | | |  |
| --- | --- | --- | --- |
| Genotype | Mutation profile | | Predition |
|  | *rpoB* 81bp core region | *katG* ∪ *inhA* promoter ∪ *ahpC* promoter |  |
|  | N | N | pan-susceptible |
|  | Y | N | RIF-resistant |
|  | N | Y | INH-resistant |
|  | Y | Y | MDR |
| Y, at least one mutation;  N, no mutation | | |  |

Table S2. Distribution of mutations in the *rpoB* gene among 227 MTB isolates from tuberculosis cases in Shenzhen, Wuhan and Shanghai, China

| Locus | Nucleotide change | No.(%) of isolates(n=227) |
| --- | --- | --- |
| *rpoB*531 | TCG→TTG | 76(33.5) |
|  | TCG→TTT | 1(0.44) |
|  |  |  |
| *rpoB*526 | CAC→CTC | 8(3.52) |
|  | CAC→TAC | 5(2.20) |
|  | CAC→GAC | 4(1.76) |
|  | CAC→CGC | 3(1.32) |
|  | CAC→CCC | 2(0.88) |
|  | CAC→ACC | 1(0.44) |
|  | CAC→TGC | 1(0.44) |
| *rpoB*526, *rpoB*535 | CAC→ACC CCC→TCC | 1(0.44) |
| *rpoB*526, *rpoB*530 | CAC→GAC CTG→CCG | 1(0.44) |
| *rpoB*526, *rpoB*518 | CAC→AAC AAC→DEL | 1(0.44) |
| *rpoB*526, *rpoB*511 | CAC→AAC CTG→CCG | 2(0.88) |
| *rpoB*526, *rpoB*509 | CAC→CGC AGC→AGA | 1(0.44) |
| *rpoB*516 | GAC→GTC | 11(4.85) |
|  | GAC→TAC | 3(1.32) |
| *rpoB*516, *rpoB*511 | GAC→GGC CTG→CCG | 2(0.88) |
| *rpoB*516, *rpoB*511 | GAC→GTC CTG→CCG | 1(0.44) |
| *rpoB*516, *rpoB*514 | GAC→GTC TTC→CTC | 1(0.44) |
| *rpoB*516, *rpoB*511, *rpoB*518 | GAC→GGC CTG→CCG AAC→GAC | 1(0.44) |
| *rpoB*511 | CTG→CCG | 4(1.76) |
| *rpoB*511, *rpoB*533 | CTG→CCG CTG→CCG | 1(0.44) |
| *rpoB*533 | CTG→CCG | 4(1.76) |
| *rpo*B533, *rpoB*530 | CTG→CCG CTG→CAG | 1(0.44) |
| *rpoB*517 | CAG→DEL | 2(0.88) |
| *rpoB*522 | TCG→TTG | 2(0.88) |
| *rpoB*513 | CAA→AAA | 1(0.44) |
| *rpoB*513, *rpoB*458 | CAA→CCA GAG→GCG | 1(0.44) |
| *rpoB*515 | ATG→GTG | 1(0.44) |
| *rpoB*527 | AAG→CAG | 1(0.44) |
| wild type |  | 83(36.6) |
| sum |  | 227(100) |

Table S3. Distribution of mutations in *katG,* promoter of *mabA-inhA* and intergenic region of *oxyR-ahpC* among 227 MTB isolates from tuberculosis cases in Shenzhen, Wuhan and Shanghai, China

| Locus | Nucleotide change | No.(%) of isolates(n=227) |
| --- | --- | --- |
| *katG*315 | AGC→ACC | 95(41.9) |
|  | AGC→AAC | 2(0.88) |
|  | AGC→ACA | 1(0.44) |
| *katG*315 , *katG*317 | AGC→AAC ATC→ACC | 1(0.44) |
| *katG* | DEL | 8(3.52) |
| *katG*273 | GGT→AGT | 1(0.44) |
| *katG*266 | ATC→ACC | 1(0.44) |
| *katG*279 , *katG*280 | GGC→DEL CCG→DEL | 1(0.44) |
| WT |  | 117(51.5) |
| SUM |  | 227(100) |
|  |  |  |
| *inhA*-15 | C→T | 21(9.25) |
| *inhA*-8 | T→A | 3(1.32) |
| *inhA* -17 | C→T | 1(0.44) |
| WT |  | 202(89.0) |
| SUM |  | 227(100) |
|  |  |  |
| *ahpC* -6 | G→A | 6(2.64) |
| *ahpC* -10 | C→T | 4(1.76) |
|  | C→A | 3(1.32) |
| *ahpC* -9 | G→A | 3(1.32) |
| *ahpC* -32 | G→A | 3(1.32) |
| *ahpC* -12 | C→A | 2(0.88) |
| *ahpC* -17 | C→T | 2(0.88) |
| *ahpC* -5 | INS T | 1(0.44) |
| *ahpC* -33 | INS | 1(0.44) |
| *ahpC* -34~-39 | DEL | 1(0.44) |
| *ahpC* -39 | C→T | 1(0.44) |
| WT |  | 200(88.1) |
| SUM |  | 227(100) |
